# Exploring the Efficacy and Target Genes of *Atractylodes Macrocephala Koidz* Against Alzheimer’s Disease Based on Multi-Omics, Computational Chemistry, and Experimental Verification

**DOI:** 10.3390/cimb47020118

**Published:** 2025-02-11

**Authors:** Yuanteng Zheng, Xin Gao, Jiyang Tang, Li Gao, Xiaotong Cui, Kechun Liu, Xiujun Zhang, Meng Jin

**Affiliations:** 1 Biology Institute, Qilu University of Technology, Shandong Academy of Sciences, 28789 East Jingshi Road, Jinan 250103, China; 2 Engineering Research Center of Zebrafish Models for Human Diseases and Drug Screening of Shandong Province, 28789 East Jingshi Road, Jinan 250103, China; 3 School of Psychology, North China University of Science and Technology, 21 Bohai Road, Tangshan 063210, China; 4 School of Public Health, North China University of Science and Technology, 21 Bohai Road, Tangshan 063210, China

**Keywords:** AMK, AD, multi-omics, computational chemistry, zebrafish

## Abstract

Objective: To unveil the efficacy and ferroptosis-related mechanisms of *Atractylodes Macrocephala Koidz* (AMK) against Alzheimer’s disease (AD), which is the most widespread neurodegenerative disease. Methods: Gene set variation analysis (GSVA) scores were used to investigate the relationship between ferroptosis and AD. Logistic regression with seven feature selections and a deep learning model were utilized to identify potential targets of AMK based on transcriptomic data from multiple tissues. A transcriptome-wide association study (TWAS), summary-data-based mendelian randomization (SMR), and mendelian randomization (MR) were utilized to validate the causal relationship between target genes and AD risk. A single-gene gene set enrichment analysis (GSEA) was employed to investigate the biological pathways associated with the target genes. Three molecular docking strategies and a molecular dynamics simulation were employed to verify the binding domains interacting with AMK. Furthermore, the anti-AD effects of AMK were validated in a zebrafish AD model by testing behavior responses, apoptosis, and the deposition of beta-amyloid (Aβ) in the brain. Ultimately, real-time qPCR was used to verify the ferroptosis-related targets, which was identified via multi-omics. Results: Ferroptosis is an important pathogenic mechanism of AD, as suggested by the GSVA scores. AMK may exert its anti-AD activity through targets genes identified in the brain (*ATP5MC3, GOT1, SAT1, EGFR*, and *MAPK9*) and blood (*G6PD, PGD, ALOX5, HMOX1,* and *ULK1*). *EGFR* and *HMOX1* were further confirmed as target genes mediating the anti-AD activity of AMK through TWAS, SMR, and MR analyses. The GSEA results indicated that *EGFR* may be involved in oxidative phosphorylation-related pathways, while *HMOX1* may be associated with lysosome and phagosome pathways. The results of three molecular docking strategies and molecular dynamics simulations implied that the kinase domain of EGFR and the catalytic domain of HMOX1 played pivotal roles in the interaction between AMK and the targets. In a zebrafish model, AD-like symptoms including motor slowness and delayed responses, neuronal apoptosis, and plaque deposition in the brain, were significantly improved after AMK treatment. Accordingly, AMK reversed the abnormal expression of *egfra* and *hmox1a*, two core targets genes involved in ferroptosis. Conclusions: AMK significantly alleviated AD-like symptoms through the modulation of EGFR and HMOX1, which might reduce lipid peroxidation, thereby suppressing ferroptosis. This study provided evidence supporting the efficacy and therapeutic targets associated with ferroptosis in AMK-treated AD, which aid in the development of therapeutic interventions.

## 1. Introduction

Alzheimer’s disease (AD) is a neurodegenerative disease marked by gradual cognitive deterioration and memory loss [1]. Furthermore, AD is the most common neurodegenerative disease, which affects more than 35 million people worldwide with an increasing tendency [2]. Although the pathogenesis of AD remains unclear, a complex interaction between genetic variation and ferroptosis is believed to underlie the disease pathogenesis [3]. Understanding the complexities of this interaction might reveal important insights into AD pathogenesis and potential targets for treatment and prevention.

In recent years, there has been increasing evidence supporting the therapeutic efficacy and advantages of traditional Chinese medicine (TCM) in AD treatment [4]. TCM demonstrated efficiency in ameliorating patient symptoms, preventing disease progression, and enhancing quality of life [5]. *Atractylodes Macrocephala Koidz* (AMK), a TCM, is a perennial herbaceous plant belonging to the Asteraceae family [6]. In traditional medicine, AMK is utilized to strengthen the spleen, eliminate dampness, and control sweating [7]. Multiple ferroptosis-related genes contribute to the complex multifactorial pathophysiology in AD [8]. For instance, genetic polymorphisms of the estrogen receptor alpha gene encoded by ESR1 are associated with ferroptosis accompanied by increased gene expression, suggesting the vital role of genetic effects on ferroptosis genes in AD [9,10]. Furthermore, the dysregulation of genes associated with ferroptosis has been observed in the hippocampus of individuals with AD [11], highlighting the association between ferroptosis and genetic variation. It is worth noting that lipid peroxidation is a key characteristic of ferroptosis [12]. The previous research suggested that AMK possesses neuroprotective activity and antioxidant activity [13], which would be beneficial to the pathology of AD. Thus, it is proposed that AMK may inhibit ferroptosis by reducing lipid peroxidation, thus ameliorating AD symptoms.

Machine learning and deep learning, as computational techniques, are greatly beneficial in the medical studies [14,15,16], aiding in the development of computer models to support the classification and prediction of new cases [17]. Genomic-related research is highly prevalent [18]. Transcriptome-wide association studies (TWAS) are widely applied to integrate gene expression data with genome-wide association studies (GWAS) to identify gene–trait associations, while fusion techniques combine different data types or models to enhance predictive power and uncover complex biological relationships [19]. Additionally, the summary data-based mendelian randomization (SMR) approach, being unaffected by confounding factors, can compensate for the limitations of transcriptomic data. The SMR method is popular in estimating the causal effect of exposure on an outcome. Molecular docking methods have been employed to validate the affinity between small molecules and proteins [20,21,22]. Mendelian randomization (MR) is a statistical analysis of genetics that can be used to predict drug efficacy by mimicking randomized controlled trials [23]. The molecular dynamics simulation approach complements the limitations of molecular docking, facilitating further exploration of the affinity between small molecules and proteins [24]. The zebrafish (Danio rerio) has become increasingly recognized for features such as external fertilization, a rapid developmental rate, and transparent embryos and larvae, increasing its range of applications. It has become an excellent model organism for studying neurodegenerative diseases, drug screenings, and drug safety evaluations. The zebrafish genome has been largely mapped, showing up to 87% homology with the human genome, with similar regulatory signaling pathways. It also holds great potential in neurobehavioral studies and is widely used to screen drugs targeting AD.

**Figure 1:**
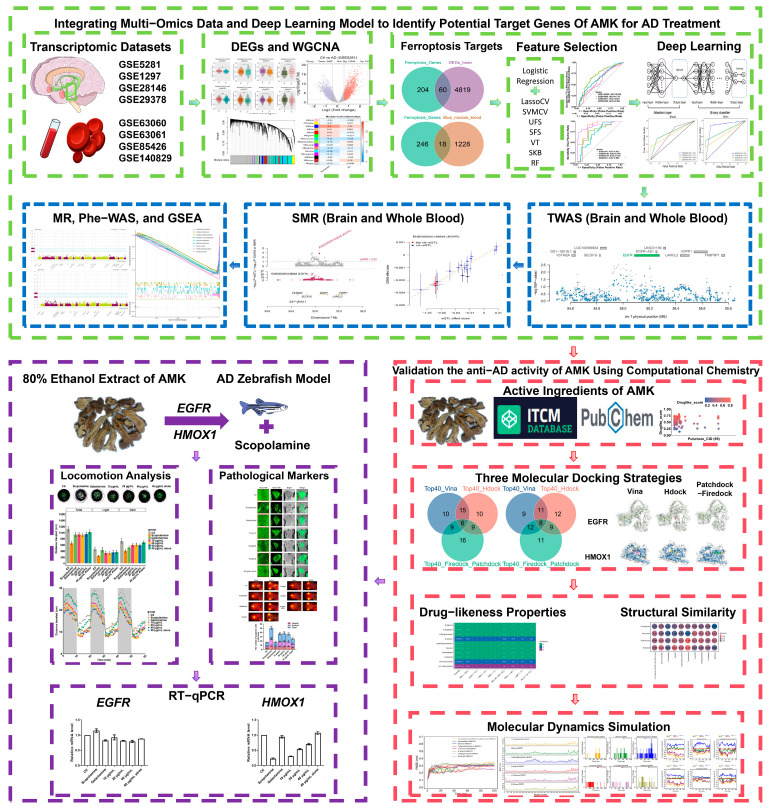
Study framework. AD—Alzheimer’s Disease, AMK—*Atractylodes Macrocephala Koidz*, DEGs—Differentially Expressed Genes, WGCNA—Weighted Gene Co-expression Network Analysis, MR—Mendelian Randomization, SMR—Standard Mendelian Randomization, *EGFR—*Epidermal Growth Factor Receptor, *HMOX1*—Heme Oxygenase-1, Phe-WAS—Phenome-Wide Association Study, GSEA—Gene Set Enrichment Analysis.

In this study, efficacy and ferroptosis-related targets for AMK-treated AD were investigated through the integration of a deep learning model and multi-omics data. Three molecular docking strategies and a molecular dynamics simulation were employed to verify the anti-AD activity of AMK. Eventually, the zebrafish AD model was used to investigate the amelioration of AD-like symptoms via AMK and the expression of ferroptosis-related targets. The study framework is shown in Figure 1.

## 2. Materials and Methods

### 2.1. Data Source

Datasets of the brain and hippocampus (GSE5281, GSE29378, GSE1297, GSE28146), as well as blood (GSE63060, GSE63061, GSE140829, GSE85426), were downloaded from the Gene Expression Omnibus (GEO) database (https://www.ncbi.nlm.nih.gov/geo/, accessed on 3 February 2025) [25]. Two GWAS datasets were included in this study. GWAS data (GCST007320) for the AD discovery cohort were obtained from the GWAS Catalog (https://www.ebi.ac.uk/gwas/home, accessed on 3 February 2025) [26], which included 71,880 clinically diagnosed or “proxy” AD cases and 383,378 controls. The AD replication GWAS cohort (ukb-b-323) was obtained from the IEU OpenGWAS project (https://gwas.mrcieu.ac.uk/, accessed on 3 February 2025) [27], which included 19,255 clinically diagnosed AD cases and 380,538 controls. Tissue expression used in the transcriptome-wide association study (TWAS) was obtained using FUSION (http://gusevlab.org/projects/fusion/, accessed on 3 February 2025). The blood cis-expression quantitative trait loci (eQTL) dataset and brain cis-eQTL dataset were used in this study. eQTLGen (https://www.eqtlgen.org/cis-eqtls.html, accessed on 3 February 2025), where the cis-eQTLs of 16,987 genes were obtained from 31,684 blood samples of healthy individuals of European ancestry [28]. Fully significant cis-eQTL results, false discovery rate (FDR < 0.05), and allele frequency information were obtained. The brain cis-eQTL data were obtained from the PsychENCODE brain consortia (https://yanglab.westlake.edu.cn/data/SMR/PsychENCODE_cis_eqtl_HCP100_summary.tar.gz, accessed on 3 February 2025) [29], which included 1387 prefrontal cortex samples, primarily European. We downloaded all significant cis-eQTLs (FDR < 0.05) for genes showing the expression of > 0.1 fragments per kilobase per million mapped fragments in at least 10 samples and all SNP information.

### 2.2. Identification of Potential Target Genes of AMK for AD Treatment Through the Integration of Multi-Omics and Logistic Regression with Seven Feature Selections and a Deep Learning Model

#### 2.2.1. Gene Set Variation Analysis (GSVA)

The GSVA is a non-parametric, unsupervised approach designed to assess the enrichment scores of transcriptomic gene sets. By comprehensively scoring the gene sets, GSVA translates gene-level changes into pathway-level changes, allowing for the assessment of biological functions in samples. A total of 264 ferroptosis-related genes were sourced from FerrDb V2 (http://www.zhounan.org/ferrdb/current/, accessed on 3 February 2025) [30]. The ferroptosis-related pathway genes, including those involved in lipid peroxidation, phosphorylation, and apoptosis, were obtained through GeneCards. The relevant targets associated with the keywords “lipid peroxidation” and “phosphorylation” were retrieved from GeneCards, with a specific focus on the biological species “Homo sapiens” [31]. In the GeneCards database, targets were filtered using a criterion of “Relevance score  ≥  20”. The ferroptosis-related pathway genes are presented in Supplementary Table S1. The “GSVA” R package was employed to compute scores for the ferroptosis gene set, allowing for the assessment of ferroptosis-related biological function changes across various samples [32].

#### 2.2.2. Analysis of Differentially Expressed Genes (Brain and Hippocampus) and WGCNA (Blood)

The “limma” R package was used to calculate differentially expressed genes (DEGs) between the Ctl and AD groups [33]. DEGs were obtained using the threshold standard |log2fold change log2FC| > 0.05, adj.*p*.Val < 0.05. The volcano plot was visualized via the “ggplot2” R packages. For a comprehensive exploration of the genes associated with ferroptosis, GSE63060 and GSE63061 were combined for a weighted gene co-expression network analysis (WGCNA). The batch effect of GSE63060 and GSE63061 was corrected using the “sva” R package [34]. WGCNA is an algorithm for the construction of gene clustering modules based on similar gene expression patterns [35]. We used the “WGCNA” R package to construct a gene co-expression network based on the merged dataset from GSE63060 and GSE63061. The gene co-expression module with the strongest positive with AD and ferroptosis were utilized for further investigation.

#### 2.2.3. Machine Learning and Feature Selection

A machine learning analysis was conducted of the brain and blood datasets using “SkLearn” Python library [36]. Logistic regression with different feature selection (FS) approaches was applied to identify potential ferroptosis-related targets. Additionally, seven FS methods (Univariate Feature Selector, SelectKBest, Variance Threshold, Sequential Feature Selection, LassoCV, SVMCV, and Random Forest) were used in this process. The parameters of the seven FS methods are demonstrated in Supplementary Table S2. The prediction performance of each classification model was estimated using *accuracy*, *recall*, *specificity*, *precision*, *ROC-AUC*, and *F1 score measures*. Their formulas are presented below:

(1) $$Accuracy=\frac{TP+TN}{TP+TN+FP+FN}$$

(2) $$Recall=\frac{TP}{TP+FN\;}$$

(3) $$Specificity=\frac{\;TN}{TN+FP}$$

(4) $$Precision=\frac{TP\;}{TP+FP}$$

(5) $$TPR=\frac{TP}{TP+FN\;}$$

(6) $$FPR=\frac{FP}{FP+TN}$$

(7) $$F1\;score=2\frac{precision\times recall}{precision+recall}$$

where *TP* = true positive; *FP* = false positive; *TN* = true negative; *FN* = false negative.

*Accuracy* is the ratio of correctly predicted subjects to the total number of subjects. The *recall* is the fraction of correctly predicted subjects related to a certain class among all subjects related to this class. *Specificity* is the proportion of true negative cases correctly identified by the classification model out of all actual negative cases. The *precision* is the fraction of correctly predicted subjects related to a certain class among all subjects for which a classification model was assigned to this class. *ROC-AUC* is the area under the error curve, which evaluates the quality of the model without being tied to a specific threshold. To building the ROC curve, the *TPR* against the *FPR* is plotted, and then the area under the resulting curve was calculated. The *F1 score* is the harmonic mean of precision and recall. We used the *F1 score*, where we averaged all per-class F1 scores.

#### 2.2.4. Deep Learning Model

An attention mechanism-binary classification model was constructed using PyTorch (Python package) to assess the discriminative power of the best feature selection integrated logistic regression on the brain and blood datasets [37]. The model included an attention layer and a basic classifier. The attention layer weighted samples to highlight relevant information and suppress irrelevant details for classification. Comprising two linear fully connected layers and a nonlinear activation function, the attention layer first mapped targets related to ferroptosis from the best feature selection model to an intermediate hidden dimension. The results were then processed through a hyperbolic tangent function before entering the second linear layer, which output a scalar value normalized using a sigmoid function to derive the attention weights.

The basic classifier utilized these weighted features for classification, employing multiple fully connected linear layers and activation functions. Input was initially processed with a dropout function to prevent overfitting, followed by ReLU activation functions in the subsequent fully connected linear layers. The final fully connected linear layer produced categorical probability values using the Softmax function.

Cross-entropy loss function measured the disparity between the model’s output probability distribution and actual labels. Model parameters were optimized using the Adam optimizer to minimize this loss function. To address data imbalances, 10-fold cross-validation was implemented with 300 epochs and a batch size of 10. Training iterations encompassed forward propagation, loss computation, back propagation, and parameter updates on the samples. Their formulas are presented below:

The linear function expression is given as follows:

(8) $$\sigma \left(X\right)=\chi$$

The ReLU (Rectified Linear Unit) function is expressed as follows:

(9) $$\left(X\right)=\left\{\begin{array}{c} \max\left(0,\chi \right),\;\chi \geq 0\\ \;\;0,\;\chi <0 \end{array}\right.$$

When the input value *χ* is greater than or equal to zero, the output is *χ* itself; when *χ* is less than zero, the output is zero.

The sigmoid function is expressed as follows:

(10) $$\sigma \left(X\right)=\frac{1}{1+e^{-\chi }\;}$$

The tanh (hyperbolic tangent) function is expressed as follows:

(11) $$\sigma \left(X\right)=\frac{2}{1+e^{-2\chi }\;}-1$$

The Softmax function is expressed as follows:

(12) $$\sigma \left(\chi_{i}\right)=\frac{e^{\chi_{i}}}{\;\Sigma_{c=1}^{C}e^{\chi_{c}}}$$

where *σ* takes values in the range (0, 1), and the sum of all class outputs equals 1. Therefore, the output of Softmax is a vector of length *C*, with the elements summing to 1. The binary cross-entropy loss is defined as follows:

(13) $$Loss=-\left[y\times {log}_{e}\left(p\right)+\left(1-y\right)\times {log}_{e}\left(1-p\right)\right]$$

#### 2.2.5. Transcriptome-Wide Association Study

FUSION is a suite of programs designed for conducting transcriptome-wide and regulome-wide association studies (TWAS and RWAS) [38]. It builds predictive models of the genetic component underlying a functional or molecular phenotype and uses GWAS summary statistics to analyze disease-associated components. In this study, a TWAS analysis was performed following the FUSION protocol with default settings.

ImpG-Summary algorithm was extended to impute the Z scores for the cis genetic component of expression.

Z: A vector representing the standardized effect sizes (z-scores) of target traits’ SNPs at a given locus.

In this study, the Z-scores for both expression and traits are modeled as a linear combination of the following elements with weights *W*.

(14) $$W=\sum_{e,s}\sum_{s,s}^{-1}$$

where

∑_*e*,*s*_: covariance matrix between all SNPs and gene expression.

∑_*s*,*s*_: covariance matrix among all SNPs (LD).

Both ∑_*e*,*s*_ and ∑_*s*,*s*_ are estimated based on a reference dataset.

(15) $$Z∽N\left(0,\sum_{s,s}\right)$$

The estimated Z-scores for expression and traits, denoted as WZ, are given by

(16) $$Var\left(WZ\right)=W\sum_{s,s}W^{t}$$

The imputed Z-scores can be obtained using the following formula:

(17) $$\frac{WZ}{{W\sum }_{s,s}W^{t^{\frac{1}{2}}}}$$

#### 2.2.6. Summary Data-Based Mendelian Randomization

We conducted an SMR analysis to evaluate the relationship between ferroptosis-related gene expression (cis-eQTL data from brain and blood) and AD risk (GWAS data) [39]. The SNPs were the instruments, brain and blood gene expression were selected as the exposure, and the AD risk was selected as the outcome of exposure (SMR *p* < 0.05, HEIDI *p* > 0.05, cis-eQTL, and GWAS *p* < 1 × 10^−5^). HEIDI *p* < 0.01 was used to detect heterogeneity in the dependent instrument. Let x represent the exposure variable (gene expression), y the outcome variable (AD risk), and z the instrumental variable (SNP). The two-stage least squares estimate of the effect of x on y is given by the following:

(18) $$\beta_{xy\;}=\frac{\beta_{zy\;}}{\beta_{zx}}$$

In this context, $\beta_{zy}$ and $\beta_{zx}$ represent the least squares estimates of y and x for z, respectively, while $\beta_{xy}$ is interpreted as the effect size of x on y, and is not confounded by non-genetic factors. The sampling variance of is given by the following:

(19) $$var\left(\beta_{xy\;}\right)=\frac{var\left(y\right)\times \left(1-R_{xy}^{2}\right)}{n\times var\left(x\right)\times R_{zx}^{2}}$$

where n is the sample size, $R_{xy}^{2}$ is the proportion of variance in explained y by x, and $R_{zx}^{2}$ is the proportion of variance in explained x by z. Therefore, the test statistic can be constructed as follows:

(20) $$T_{MR}=\frac{\beta_{xy\;}}{var\left(\beta_{xy\;}\right)}$$

which is used to test the significance of $\beta_{xy}$, with TMR∼$\chi_{1}^{2}$.

#### 2.2.7. Mendelian Randomization

An MR analysis was conducted using the “TwoSampleMR” R package [37]. Prior to the MR analysis, a rigorous quality control of the SNP instrumental variables was performed. F-statistics and R^2^ were used to select instrumental variables with strong effects. The specific formulas are as follows:

(21) $$R^{2}=2\times MAF\times \left(1-\;MAF\right)\times \beta^{2}$$

(22) $$F=\frac{N-\;K-1}{K}\times \frac{R^{2}}{1{-R}^{2}}$$

Here, $R^{2}$ represents the proportion of variance in the exposure explained by the SNPs, where MAF denotes the minor allele frequency, and *β* is the effect size of the allele. *N* refers to the sample size of the exposure, and *K* is the number of SNPs. SNPs were required to have an *F* ≥ 10. Variants were carefully selected based on their low linkage disequilibrium (LD, *r*^2^ < 0.1) using the 1000 Genomes European panel. Additionally, SNPs whose variance contribution to the outcome exceeded their contribution to the exposure, as determined using the Steiger filtering method, were excluded.

In the primary analysis, meta-analyses were conducted using the inverse-variance weighted (IVW) method, the weighted median method, and the MR-Egger method to obtain the SNP effect estimates. The Wald ratio estimate was also calculated for each SNP. The IVW method provides the highest statistical power under the assumption that all instrumental variables are valid. When the results of the three MR methods were inconsistent, the IVW method was prioritized as the main result. The weighted median MR method calculates the median of the SNP-specific estimates, weighted by their precision, and can tolerate up to 50% invalid instruments. Standard errors were calculated using the bootstrap method. This method is robust to horizontal pleiotropy, which refers to situations where certain SNPs affect the outcome through pathways other than the exposure. Although the MR-Egger method can also evaluate horizontal pleiotropy, its statistical power is lower compared to the IVW method. When all three methods yielded consistent estimates, this indicated that horizontal pleiotropy did not significantly bias the IVW results. Cochran’s *Q* and the MR-Egger intercept test were used to assess horizontal pleiotropy and heterogeneity for genetic instruments with multiple SNPs.

#### 2.2.8. Phenome-Wide Association Study (Phe-WAS)

To study the potential side effects of drug target genes from brain and blood, we conducted a Phe-WAS analysis of the AstraZeneca PheWAS Portal (https://azphewas.com/, accessed on 3 February 2025) [40]. Phe-WAS analysis is a study design that tests the associations of a given SNPs or gene with numerous different phenotypes, providing a complementary approach to GWAS. It has proved useful in recovering previously detected genotype–phenotype associations and in discovering new ones.

#### 2.2.9. Single-Gene Gene Set Enrichment Analysis (GSEA) Analysis

Based on the key target genes (*EGFR* and *HMOX1*), the merged dataset samples were divided into high- and low-expression groups according to their median expression levels. Subsequently, a single-gene GSEA was performed using the “clusterProfiler” and “org.Hs.eg.db” R packages to identify enriched regulatory pathways and the biological functions of the high- and low-expression groups. The enrichment score (ES), normalized enrichment score (NES), *p*-value, and adjusted *p*-value were calculated based on 1000 permutations. A positive ES suggests that the gene set is activated, whereas a negative ES indicates its suppression. The selection criteria included adjusted *p*-value < 0.05. Finally, the top 10 most significant results for KEGG enrichment were visualized separately.

### 2.3. Validation of the Anti-AD Activity of AMK Using Computational Chemistry

#### 2.3.1. Molecular Docking

The PDB files of proteins (EGFR and HMOX1) were downloaded from UniProt (https://www.uniprot.org/, accessed on 3 February 2025) [41]. The 3D molecular structures of AMK ingredients were obtained from the PubChem database (https://pubchem.ncbi.nlm.nih.gov/, accessed on 3 February 2025) [42]. To identify key ingredients of AMK, three molecular docking strategies were followed: AutoDock Vina, HDOCK, and PatchDock-FireDock. These molecular docking strategies allow for mutual validation. AutoDock Vina, using a Lamarckian genetic algorithm, was utilized for a molecular docking investigation [43]. We used HDOCK (http://hdock.phys.hust.edu.cn/, accessed on 3 February 2025), a hybrid algorithm combining template-based modeling and ab initio free docking, to perform molecular docking [44]. PatchDock and FireDock were utilized in a two-tier approach to predict the receptor–ligand complexes. The PatchDock algorithm (https://bioinfo3d.cs.tau.ac.il/PatchDock/, accessed on 3 February 2025), which relies on shape-complementarity molecular shape representation, surface patch matching, filtering, and scoring, was used for the initial docking predictions [45]. The FireDock algorithm (https://bioinfo3d.cs.tau.ac.il/FireDock/, accessed on 3 February 2025) allows for fast interaction refinement in molecular docking and was used for refinement, including the optimization of side-chain conformations, rigid-body orientation, and the rescoring of the initial docking solutions [46].

#### 2.3.2. Drug-likeness Properties and Structural Similarities

To ensure the efficacy of the potential targets, we screened the ingredients of AMK with Lipinski’s rule of five. The drug-likeness characteristics of AMK were evaluated using the SwissADME (https://www.swissadme.ch/, accessed on 3 February 2025) [47]. Clinical drug selection: Rivastigmine, memantine, galantamine, and donepezil were analyzed by generating ECFP fingerprints using the RDKit library (Python) and calculating Tanimoto coefficients to perform a clinical drug similarity screening [48].

#### 2.3.3. Molecular Dynamics (MD) Simulation

The GROMACS software (2023) was utilized to perform atomistic MD simulations. To validate the results of the molecular docking, a combination of the three methods was used to conduct the MD simulations for 100 ns. The PDB files of proteins (EGFR and HMOX1) were processed to construct the relevant topology structure; then, the topology structure of the ligand was processed using the ACPYPE web server. The ligand information was then incorporated into the protein topology file to generate a complex file. The AMBER99SB-ILDN force field and three-point transferable intermolecular potential (TIP3P) water model were utilized for calculations, and the system was confined within a cubic box with dimensions of 1.0. An SPC216 water solvent and Na^+^/Cl^−^ ions were added to neutralize the charge and prevent collisions between the protein and the ligand. The complex systems were relaxed using the steepest descent and then gradually heated to 300 K with constraints. The Particle Mesh Ewald PME method was employed to compute short-range electrostatic and van der Waals interactions, ensuring the accuracy and stability of the simulations. To maintain the temperature of the system at 300 K, the V-rescale temperature coupling method was used. Similarly, the Parrinello–Rahman method was applied to maintain constant pressure at 1 bar. Then, the position restraint simulation of 50,000 steps (2 fs each step) was implemented under NVT and NPT conditions. The complex systems were finally subjected to a 100 ns MD simulation with a 2 fs time step, and the trajectory data were saved every 10 ps. The simulation results were visualized using vmd 1.9.4.

#### 2.3.4. Binding-Free Energy Calculation

The binding-free energy of the systems was calculated using the Molecular Mechanics/Poisson–Boltzmann Surface Area (MM/PBSA) approach, executed with gmx_MMPBSA. The calculation was performed using the following formula:

(23) $$G_{bind}={\Delta G}_{complex}-\left({\Delta G}_{receptor}+\Delta G_{ligand}\right)$$

where Δ*G_bind_* is the total binding energy, Δ*G_complex_* is the binding energy of the free complex, Δ*G_receptor_* is the binding energy of the free receptor, and Δ*G_ligand_* is the binding free energy the of unbounded ligand.

The above equations can also be expressed as follows:

(24) $$G_{bind}=\Delta H-T\Delta S$$

where Δ*H* represents the enthalpy of the ligand binding, and −*T*Δ*S* represents the configurational entropy of the ligand upon binding. When the entropy term is neglected, the computed value corresponds to the effective free energy, which is typically sufficient to compare the relative binding free energies of related ligands.

Δ*H* can be decomposed into different terms:

(25) $$\Delta H={\Delta E}_{mm}+{\Delta G}_{sol}$$

where Δ*E*mm refers to the change in molecular mechanics energy in the gas phase, and Δ*G*sol refers to the molecular mechanics energy change in the solvent.

Specifically,

(26) $${\Delta E}_{mm}={\Delta E}_{bonded}+{\Delta E}_{nonbonded}$$

(27) $${\Delta E}_{mm}=\left({\Delta E}_{bond}+{\Delta E}_{angle}+{\Delta E}_{dihedral}\right)+\left({\Delta E}_{ele}+{\Delta E}_{vdW}\right)$$

where Δ*E*_*mm*_ includes the internal energy (Δ*E*_*bonded*_) and the non-bonded interaction energy (Δ*E*_*nonbonded*_). The internal energy consists of the bond energy (Δ*E*_*bond*_), angle strain energy (Δ*E*_angle_), and torsional energy (Δ*E*_*dihedral*_), while the non-bonded interaction energy includes contributions from van der Waals interactions (Δ*E*_*ele*_) and electrostatic interactions (Δ*E*_*vdW*_).

Additionally,

(28) $${\Delta G}_{sol}={\Delta G}_{polar}+{\Delta G}_{non-polar}={\Delta G}_{\frac{PB}{GB}}+{\Delta G}_{non-polar}$$

where Δ*G*_*polar*_ represents the polar component of the solvation energy and Δ*G*_*non-polar*_ represents the non-polar component. The PB (Poisson–Boltzmann) model and GB (Generalized Born) model estimate only the polar component of the solvation process.

### 2.4. Validation of AMK’s Potential Use in Ameliorating AD-like Symptoms and Ferroptosis-Related Target Expression in a Zebrafish Model

#### 2.4.1. Materials, Reagents and Animals

AMK was purchased from Zhejiang (Shaoxing China) and Scopolamine was purchased from Sigma-Aldrich (St. Louis, MO, USA). Zebrafish of wild-type AB strain were maintained as per the standard protocol [49]. Zebrafish were maintained at 28 °C (under 14 h light/10 h dark conditions). Zebrafish embryos were obtained via natural mating, cleaned, and preserved in bathing medium containing 2 mg/L methylene blue. Embryos from 6 (dpf) days past fertilization were examined under a dissecting microscope (Olympus, Tokyo, Japan) and only those embryos that developed to the blastula stage were used for further experimentations.

#### 2.4.2. Scopolamine-Induced AD Model

Zebrafish embryos at 1 dpf were dechorionated manually and transferred randomly to 6-well cell culture plates containing 20 eggs per well with 4 mL of bathing medium. Subsequently, embryos were exposed to scopolamine at 6 dpf to generate AD-like symptoms, as per previously reported studies [50]. Related research indicated that scopolamine is capable of producing AD-like symptoms [51]. The concentration of 1000 μM was selected to induce the zebrafish AD model.

#### 2.4.3. AMK Preparation

The dried roots of AMK were ground into a fine powder. Approximately 50 g of the powder was placed in a 1000 mL round-bottom flask with 350 mL of distilled water, and an essential oil extractor was installed for steam distillation to extract the essential oil, which was then collected. The water-boiled solution was concentrated under reduced pressure, followed by separation using macroporous resin D101 or AB-8 type with either a water–ethanol gradient elution or gradient solvent extraction with petroleum ether, ethyl acetate, and n-butanol. Additionally, AMK powder was extracted using ultrasound-assisted extraction with 80% ethanol at 15 times the sample volume; this was repeated three times for 2 h each. The combined extracts were centrifuged at 5000 rpm for 15 min to collect the supernatant, which was then concentrated using rotary evaporation under reduced pressure. Ethanol was slowly added to the solution until a concentration of 80% was reached. This solution was kept at 4 °C for 24 h to allow for precipitation. After centrifugation to remove macromolecular impurities, the final product was freeze-dried to obtain AMK extract powder.

#### 2.4.4. AMK Treatment

Zebrafish were co-treated with scopolamine and different concentrations of AMK ranging from 1 to 6 dpf to elucidate the neuroprotective effect of AMK. Three concentrations of AMK (10, 20, and 40 μg/mL) were selected to investigate its anti-AD activity. These concentrations were determined according to the LC50 mortality curve, with the LC50 results provided in Supplementary Figure S1. AMK at these dosages did not cause any obvious side effects. Additionally, we selected galantamine as a positive control drug for a comparison of the amelioration of AD-like symptoms [52].

#### 2.4.5. Locomotion Analysis

The behavioral tracks of zebrafish were recorded after exposure to scopolamine for 6 dpf. To avoid the effect of malformation on behavioral performance, larval zebrafish without apparent defects were selected for locomotion recording. In addition, we performed a light/dark challenge to investigate the neuroprotective effect of AMK on the locomotor ability of zebrafish. We placed zebrafish in 48-well plates, one per well, with 1 mL of bathing medium per well. After a 15 min acclimatization period, we used a Viewpoint video tracking system (Lyon, France) to record zebrafish locomotor activity at 28 ± 0.5 °C. The light source was turned off during general locomotion. Zebrafish measured for locomotion responses were then collected and assayed by real-time qPCR, along with those with obvious defects. In the 100% light/dark with lights off challenge, the test time was 60 min and the test consisted of three cycles of light/dark phases lasting 10 min per phase [53,54]. Digital data were analyzed using Viewpoint Zeblab software 3.3 (Lyon, France) to analyze the digital trajectories. There were twenty-four zebrafish larvae per group (*n* = 24).

#### 2.4.6. Apoptosis Assessment

Apoptosis in brain tissue was evaluated using the TUNEL Apoptosis Assay Kit (Beyotime, Shanghai, China). Briefly, zebrafish larvae at 6 days post-fertilization (dpf) were fixed in 4% paraformaldehyde. Following fixation, the larvae were incubated at room temperature for 15 min in a blocking solution containing 3% hydrogen peroxide in methanol. The larvae were then treated with the TUNEL reaction mixture. Fluorescent images were captured using a fluorescence microscope (Zeiss, Jena, Germany), and the fluorescence intensity of apoptotic cells in the head region was quantified using Image-Pro Plus version 5.1.

#### 2.4.7. Detection of Aβ Deposition

Zebrafish larvae were fixed in 4% paraformaldehyde and subsequently embedded in standard agar blocks. These blocks were then frozen at −20 °C until sectioning at 7 µm intervals. The tissue sections were stained with thioflavin S to detect Aβ deposition. The sections were washed several times with 0.01 M phosphate-buffered saline (PBS), with 5–10 min per wash, at room temperature. The sections were then incubated overnight in the dark at room temperature with a 0.3% solution of thioflavin S (Sigma-Aldrich, Darmstadt, Germany). After staining, the sections were washed with 0.01 M PBS for 30 min in the dark and analyzed using confocal inverted microscopy.

#### 2.4.8. RNA Extraction and Real-Time qPCR

The expression of potential genes (*EGFR*, *HMOX1)* was detected using real-time qPCR. The zebrafish larvae (*n =* 30) were collected for 6 days. The total RNA was extracted under the manufacturer’s protocol of EASY spin Plus RNA Mini Kit (RN2802; Aidlab Biotechnologies; Beijing, China). The total RNA was extracted under the manufacturer’s protocol of EASY spin Plus RNA Mini Kit (RN2802; Aidlab Biotechnologies; Beijing, China). Real-time qPCR was performed using SYBR^®^ Premix DimerEraser™ (Takara, Tokyo, Japan) and the Light Cycler^®^ 96 System (Light Cycler^®^ Instrument; Roche. Switzerland). All procedures were performed according to the manufacturer’s instructions. The mRNA levels of the target genes were standardized using the housekeeping gene *rpl13a* and the real-time qPCR reactions were performed with three replicates. The primer sequence information is presented as follows: *egfra*-RT-F: AACGCAAATAATGGCAGGAC; *egfra*-RT-R: TCTCCAGAACCACAGTGCAG; *hmox1a-*RT-F: CACGCTTACACCCGCTACCT; *hmox1a*-RT-R: ATGCTGCTTCATTTCCTTTATCAC.

### 2.5. Statistical Analysis

Python (3.7.12) and R (4.3.2) programs were utilized for statistical analysis. The Wilcoxon signed-rank test was carried out to obtain statistical comparisons between the control and AD groups. * p < 0.05 indicates statistical significance. ** p < 0.01, *** p < 0.001 or **** p < 0.0001 indicates extreme significance. Data from the experiments were analyzed using one-way ANOVA followed by Dunnett’s post-hoc test, utilizing Graph Pad Prism 8.0 (GraphPad Software; La Jolla, CA, USA). The results were expressed as mean ± sem.

## 3. Results

### 3.1. The Ferroptosis-Related Targets from Brain and Blood

**Figure 2:**
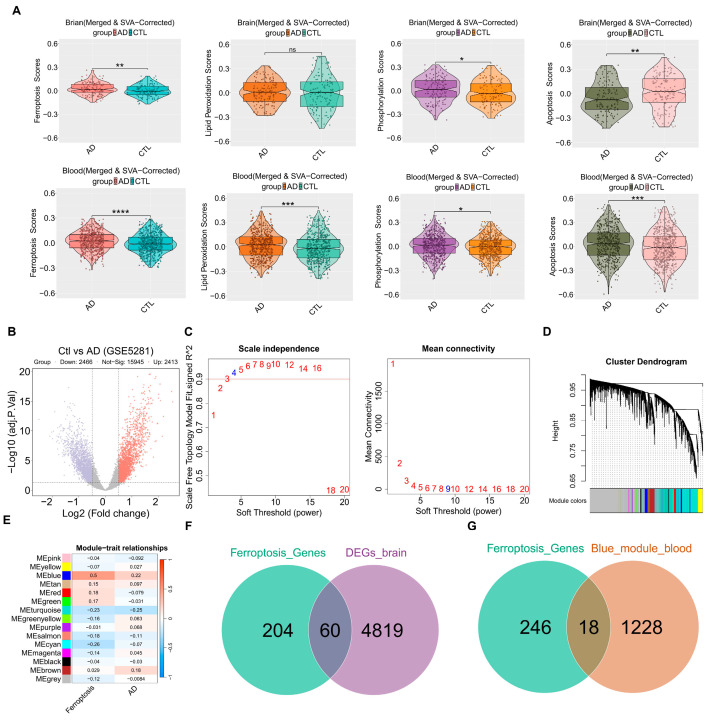
The ferroptosis-related targets from the brain and blood. (**A**) The Violin plots of GSVA ferroptosis and related pathways scores between the control group and AD group in each dataset. (**B**) The DEGs volcano plot comparing control and AD groups. (**C**) Analysis of scale-free fitting index for soft-thresholding powers. (**D**) Cluster dendrogram. (**E**) Module–trait correlation heatmap: red represents a positive correlation; blue represents a negative correlation. (**F**) Venn showing the intersection between ferroptosis genes and DEGs. (**G**) Venn showing the intersection between ferroptosis genes and the blue module. * *p* < 0.05; ** *p* < 0.01; *** *p* < 0.001; **** *p* < 0.0001; ns—not significant; AD vs. CTL. AD—Alzheimer’s Disease Group, Ctl—Control Group, DEGs—Differentially Expressed Genes, SVA—Set Variation Analysis.

After combining the brain (GSE5281, GSE29378, GSE1297, GSE28146) and blood (GSE63060, GSE63061, GSE140829, GSE85426) datasets, batch correction was performed using the “SVA” R package. To investigate ferroptosis and related pathways, the pathway activity scores were calculated using the GSVA method. In the brain tissue, significant differences were observed in the scores of ferroptosis, phosphorylation, and apoptosis, suggesting that these pathways play a major role. In the blood, all four of the above-mentioned bioprocesses showed significant differences, with the ferroptosis pathway being particularly active (Figure 2A). “limma” package was utilized to analyze DEGs in the GSE5281 dataset. The analysis identified 4879 genes that were differentially expressed between the control and AD groups, based on the criteria of |log2FC| > 0.05 and adj.*p*.Val < 0.05. In this study, 2413 genes were upregulated and 2466 genes were downregulated, as shown in Figure 2B. Detailed lists of these genes can be found in Supplementary Table S3. To investigate the role of genes related to ferroptosis in AD patients, the ferroptosis scores and AD groups were used as feature data for the WGCNA analysis. We transformed the variable AD groups into dummy variables. A scale-free topological network (β = 4) was constructed (Figure 2C), and a hierarchical clustering tree grouped highly co-expressed genes into modules, color-coded accordingly (Figure 2D). Subsequently, the correlation analysis generated a heatmap depicting module–trait relationships, revealing that the blue module was significantly correlated with ferroptosis and AD (Figure 2E). The genes included in the blue module are shown in Supplementary Table S4. A total of 1246 genes (blue module) were considered as targets related to ferroptosis in blood. Ultimately, the intersection of ferroptosis genes and DEGs was identified, obtaining 60 candidate genes that could serve as potential targets in the brain (Figure 2F). Similarly, the intersection of ferroptosis genes and the blue module was determined, obtaining 18 candidate genes as potential targets in the blood (Figure 2G). The potential targets associated with ferroptosis are presented in Supplementary Table S5.

### 3.2. Feature Selection and Deep Learning Model Validation

**Figure 3:**
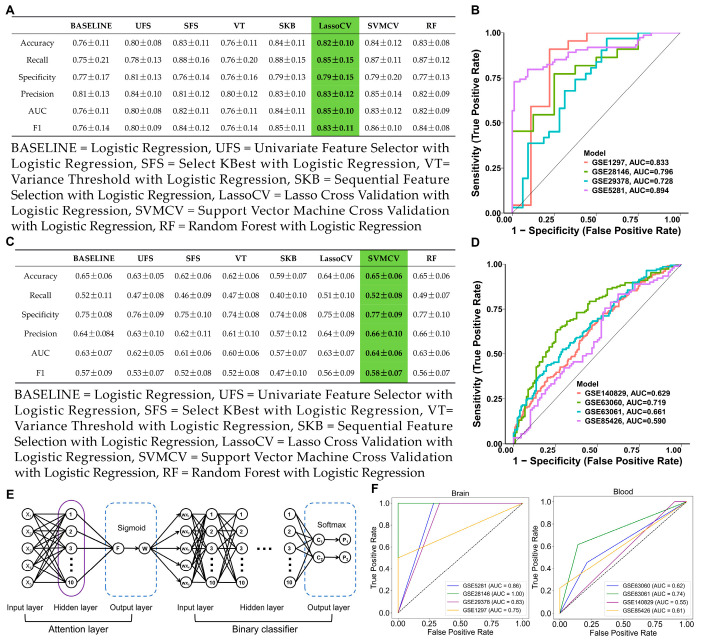
Feature selection and deep learning model. (**A**) The performance of the seven FS methods and baseline model in the GSE5281 dataset. (**B**) ROC curves of LassoCV in brain datasets. (Green represented the method with the best performance) (**C**) The performance of the seven FS methods and baseline model in the integrated (GSE63060 and GSE63061) dataset. (Green represented the method with the best performance) (**D**) ROC curves of SVMCV in blood datasets. (**E**) The neural network architecture of the attention mechanism–binary classification algorithm. (**F**) ROC curves of the deep learning model in the brain and blood datasets.

To further identify potential targets, seven FS methods with logistic regression were used in the brain (GSE5281) and integrated blood (GSE63060 and GSE63061) datasets. In the GSE5281 dataset, the LassoCV method with logistic regression was the best-performing method (F1 = 0.83 ± 0.11; AUC = 0.85 ± 0.10), as shown in Figure 3A. The features of seven FS methods with logistic regression in GSE5281 are shown in Supplementary Table S6. To validate the generalization capacity of LassoCV method, the ROC curves showed an AUC ranging from 0.72 to 0.89 in the brain datasets, suggesting that the LassoCV method with the logistic regression model corroborated their promising generalization capacity (Figure 3B). In the integrated blood GSE63060 and GSE63061 dataset, SVMCV method with logistic regression was the best-performing method (F1 = 0.58 ± 0.07; AUC = 0.64 ± 0.06) as shown in Figure 3C. The features of seven FS methods with logistic regression in the integrated blood (GSE63060 and GSE63061) dataset are shown in Supplementary Table S7. Similarly, the ROC curves showed an AUC ranging from 0.59 to 0.71 in the blood datasets, indicating that the SVMCV method with the logistic regression model demonstrated a degree of generalization ability (Figure 3D). Eventually, the attention mechanism-based deep learning model was used to validate potential targets in the brain and blood datasets using the top five features as inputs (Figure 3E). In brain tissue genes, the top five features were *ATP5MC3*, *GOT1, SAT1*, *EGFR*, and *MAPK9*. In blood tissue genes, the top five features were *G6PD*, *PGD, ALOX5*, *HMOX1*, and *ULK1*. The attention-based deep learning model could improve gene feature representation, enhance discriminative power, and strengthen the AUC’s reliability and the model generalization ability. Therefore, we employed this model to assess the reliability and efficacy of the selected target genes. Notably, the deep learning model led to improvements in the brain datasets and blood datasets, indicating that the selected features demonstrated a strong discriminative power and model generalization ability (Figure 3F). Compared with potential targets in the blood, the potential targets in the brain exhibited higher and more stable AUC values.

### 3.3. Identification of Potential Drug Target Genes in Brain and Blood Using TWAS, SMR, and MR

**Figure 4:**
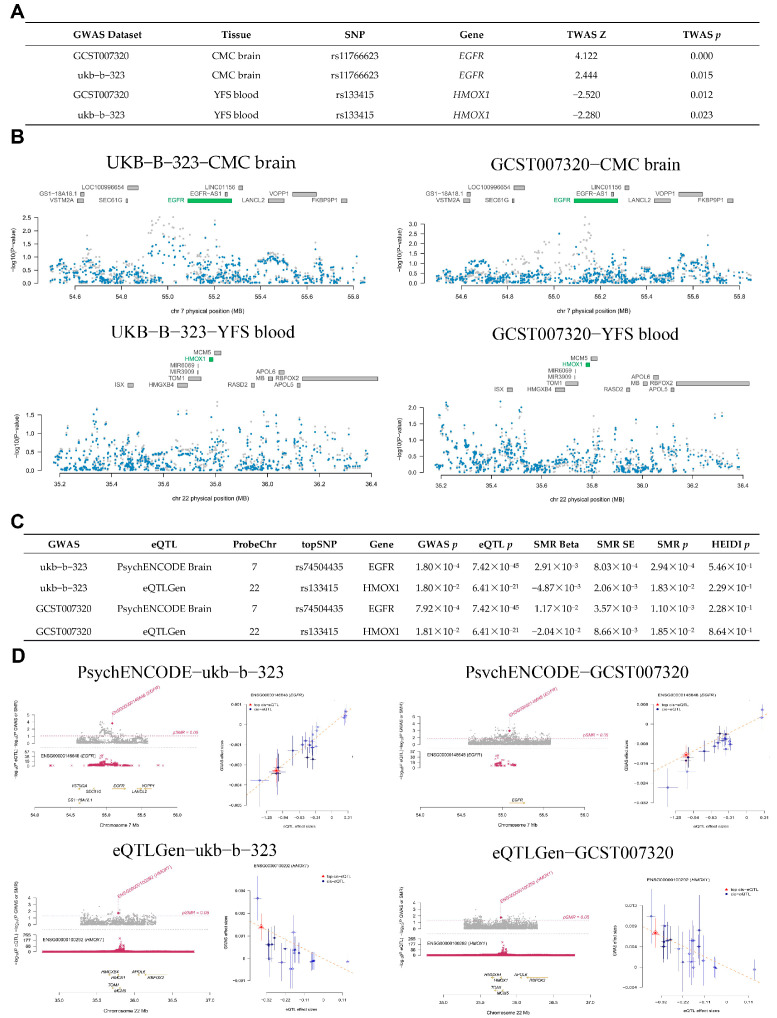
The potential drug target genes in the brain and blood, identified using TWAS and SMR. (**A**) The results of the TWAS study. (**B**) Visualization of the TWAS results. (**C**) SMR association between gene expression (brain and blood) and overall risk of AD. (**D**) Visualization of SMR results. SNP—Single Nucleotide Polymorphism, TWAS—Transcriptome-Wide Association Studies.

Based on brain (A*TP5MC3*, *GOT1*, *SAT1*, *EGFR*, and *MAPK9*) and blood (*G6PD*, *PGD*, *ALOX5*, *HMOX1*, and *ULK1*) features, a TWAS analysis was conducted in conjunction with AD GWAS (GCST007320 and ukb-b-323) to investigate genes associated with the risk of AD. The TWAS was conducted to explore the associations between ferroptosis-related targets and AD risk. In brain tissue genes, *EGFR* was significantly associated with AD risk in both analyses (TWAS *p* < 0.05), with a positive correlation between *EGFR* gene expression and AD risk (TWAS Z = 4.122, 2.444) (Figure 4A). In blood tissue genes, *HMOX1* was also significantly associated with AD in both analyses (TWAS *p* < 0.05), showing a negative correlation between *HMOX1* gene expression and AD risk (TWAS Z = −2.520, −2.280) (Figure 4A). A visualization of the TWAS results is shown in Figure 4B.

Based on cis-eQTL (*EGFR*, *HMOX1*) data from the brain and blood, along with AD GWAS, we utilized SMR to investigate the potential causal relationship between genes and AD risk. The results of the SMR analysis indicated the potential causal role of two genes (*EGFR* and *HMOX1*) in AD risk (SMR *p* < 0.05, HEIDI *p* > 0.05), as shown in Figure 4C. The gene expression of *EGFR* was positively associated with AD risk (SMR Beta = 2.91 × 10^−3^, 1.17 × 10^−2^), while the gene expression of *HMOX1* was negatively correlated with AD risk (SMR Beta = −4.87 × 10^−3^, −2.04 × 10^−2^), as shown in Figure 4C. The SMR analysis is shown in Figure 4D. Recent research revealed that the sensitivity of ferroptosis is also affected by EGFR or mutated IDH1 [55]. Additionally, non-canonical ferroptosis is triggered by excessive HMOX1 activity [56]. Thus, the *EGFR* and *HMOX1* may be associated with ferroptosis, making them suitable drug target genes for AD treatment.

A total of 202 cis-eQTLs related to the *EGFR* gene were extracted from the PsychENCODE dataset (Supplementary Table S8), with the selection criterion of a *p*-value less than 1 × 10^−6^, and a linkage disequilibrium (LD)-based screening method was applied. Specifically, an r^2^ threshold of 0.001 and a window size of 10 kb were used. Following this, the clumping method was applied to cluster these cis-eQTLs in order to remove highly correlated SNPs and ensure that the LD between the selected SNPs was low. After clumping, 29 cis-eQTLs associated with *EGFR* were retained for further analysis. Subsequently, the F-statistic and R^2^ values for each SNP were calculated, and a threshold of F > 10 was used for filtering to ensure that the included SNPs had a high explanatory power. Finally, 29 *EGFR*-related cis-eQTLs were selected for further analysis. In addition, cis-eQTL data (70) for the *HMOX1* genes were extracted from the CAGE BLOOD database (Supplementary Table S8), using similar methods. As a result, 61 cis-eQTLs related to the *HMOX1* gene were obtained.

A Mendelian randomization analysis was performed for the cis-eQTLs related to *EGFR* and *HMOX1* in the GCST007320 and ukb-b-323 datasets, with the results presented in Table 1. In both analyses, all three methods indicated a causal association between the *EGFR* gene and AD risk (*p* < 0.05), suggesting that the upregulation of EGFR expression increases the risk of AD. Similarly, all three methods, in both analyses, indicated a causal association between the *HMOX1* gene and AD risk (*p* < 0.05), with the downregulation of *HMOX1* expression increasing AD risk. No significant heterogeneity (*p* > 0.05, Table 2) or horizontal pleiotropy (*p* > 0.05, Table 3) was found for *EGFR* and *HMOX1* genes in both analyses.

### 3.4. Phe-WAS and GSEA

**Figure 5:**
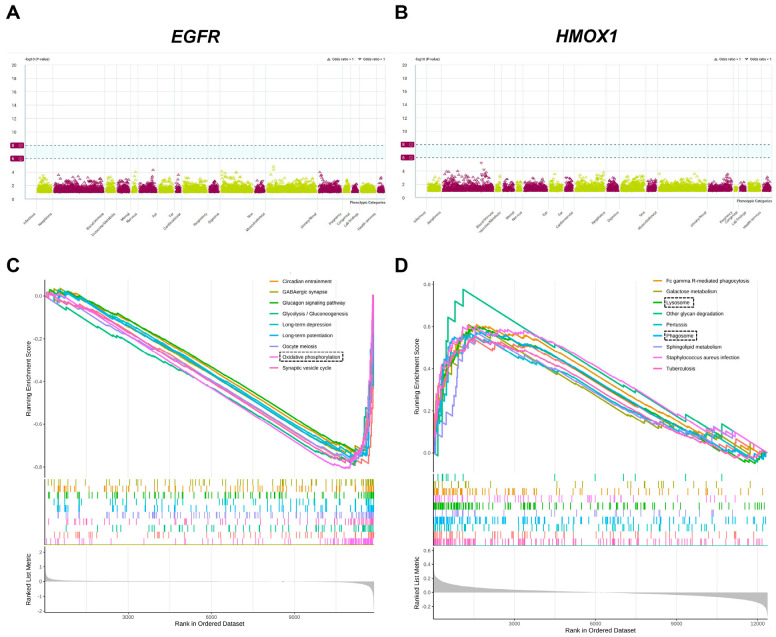
Phe-WAS and GSEA. Target Genes: Epidermal Growth Factor Receptor (EGFR); Heme Oxygenase 1 (HMOX1). (**A**) The Phe-WAS result of EGFR. (**B**) The Phe-WAS result of HMOX1. (**C**) The GSEA result of EGFR. (**D**) The GSEA result of HMOX1. *EGFR*—Epidermal Growth Factor Re-ceptor, *HMOX1*—Heme Oxygenase-1.

To evaluate the potential side effects of *EGFR* and *HMOX1*, we performed a Phe-WAS analysis. This study utilized 17,361 binary phenotypes and 1419 quantitative phenotypes from the AstraZeneca PheWAS Portal database [57]. The Phe-WAS results can be interpreted as the association between genetically determined protein expression and specific diseases or traits. There were no significant side effects associated with *EGFR* and *HMOX1* (*p* < 5 × 10^−8^) (Figure 5A,B). In the brain dataset, the downregulation of *EGFR* may be involved in oxidative phosphorylation-related pathways, suggesting that *EGFR* potentially regulates oxidative phosphorylation to reduce the accumulation of Aβ (Figure 5C). In the blood dataset, the upregulation of *HMOX1* is highly associated with lysosome- and phagosome-related pathways, suggesting that *HMOX1* may activate autophagy. Following the activation of autophagy, cellular apoptosis and amyloid protein accumulation could be improved (Figure 5D).

### 3.5. Three Molecular Docking Strategies, Drug-likeness Properties, and Structural Similarities

**Figure 6:**
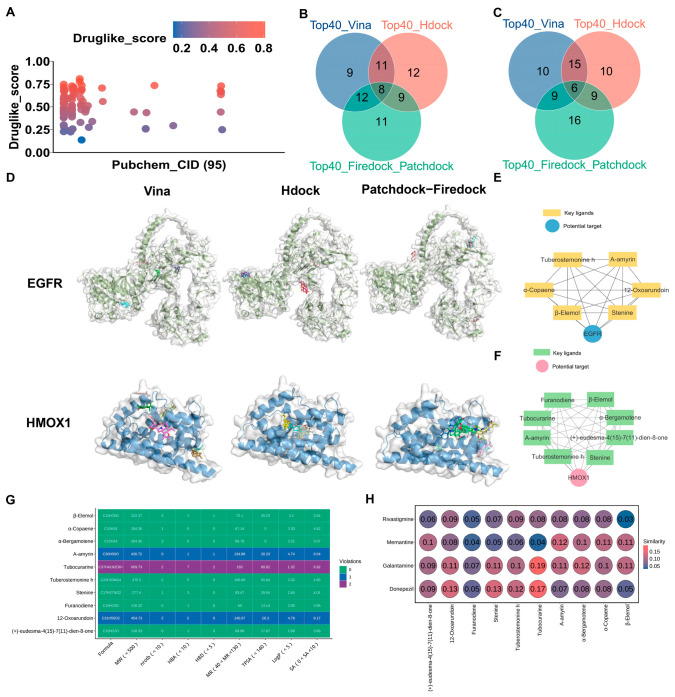
Three molecular docking strategies, drug-likeness properties, and structural similarities. (**A**) The main active ingredients of AMK from the ITCM database. (**B**,**C**) Venn diagram of the intersection of three molecular docking strategies for binding with EGFR and HMOX1. (**D**) A visualization of the intersection of three molecular docking strategies. (**E**,**F**) The network of six AMK ingredients with EGFR and eight AMK ingredients with HMOX1. (**G**) The drug-likeness properties and developability of the active AMK ingredients. (**H**) Structural similarities between the main active ingredients of AMK and clinical drugs. *EGFR—*Epidermal Growth Factor Receptor, *HMOX1*—Heme Oxygenase-1.

To verify the anti-AD activity of AMK, three molecular docking strategies were utilized. The main ingredients of AMK, totaling 95, were obtained from ITCM database (Figure 6A). The information regarding the AMK ingredients is presented in Supplementary Table S9. The 95 active ingredients of AMK were docked with EGFR and HMOX1 using three molecular docking strategies. The intersection of the three molecular docking strategies (top 40) were considered key ingredients in AMK’s binding with drug targets (Figure 6B,C). The results of the three molecular docking strategies are displayed in Supplementary Tables S10–S12. A visualization of the three molecular docking strategies is shown in Figure 6D. Specifically, six AMK ingredients were identified as interacting with EGFR, including Tuberostemonine h, A-amyrin, 12-Oxoarundoin, Stenine, β-Elemol, and α-Copaene. Eight AMK ingredients were identified as interacting with HMOX1, including Furanodiene, β-Elemol, α-Bergamotene, (+)-eudesma-4(15)-7(11)-dien-8-one, Stenine, Tuberostemonine h, A-amyrin, and Tubocurarine. The networks of ingredients involved in AMK binding with drug targets are illustrated in Figure 6E,F.

Lipinski’s rule of five is one of the key factors required for a compound to become a drug. After integrating six ingredients with EGFR and eight ingredients with HMOX1, ten AMK ingredients were selected to analyze the properties. The following criteria were considered in the drug-likeness prediction, including molecular weight < 500, number of hydrogen acceptors < 5, number of hydrogen donors < 10, Log*p* < 5, molar reflectivity (MR) range from 40 to 130. As shown in Figure 6G, Tubocurarine did not meet the criterion of molecular weight < 500, while nine AMK ingredients had an MW below 500. For number of hydrogen acceptors, Tubocurarine failed to satisfy the requirement of < 5. Additionally, 10 AMK ingredients had fewer than ten hydrogen donors. All AMK ingredients had a Log*p* below 5. 12-Oxoarundoin, A-amyrin, and Tubocurarine did not meet the criterion of an MR range from 40 to 130. When the violation count is less than or equal to 2, this indicates that the compound demonstrates favorable drug-likeness properties. Therefore, all active AMK ingredients exhibited promising drug-like properties. Moreover, three indicators, including number of rotatable bonds nrotb < 10, topological polar surface area (TPSA) < 140, and synthetic accessibility (SA) ranges from 0 to 10, were used to assess the developability of the AMK ingredients (Figure 6G). Therefore, all AMK ingredients exhibited potential for development. As shown in Figure 6H, Tubocurarine, 12-Oxoarundoin, and (+)-eudesma-4(15)-7(11)-dien-8-one exhibited high structural similarity to four clinical drugs: Donepezil, Galantamine, Rivastigmine, and Memantine.

### 3.6. MD Simulation and MM-PBSA Energy Calculations

To validate the results of the molecular docking, 100 ns MD simulations were conducted simultaneously for six AMK ingredients with EGFR and eight AMK ingredients with HMOX1.

**Figure 7:**
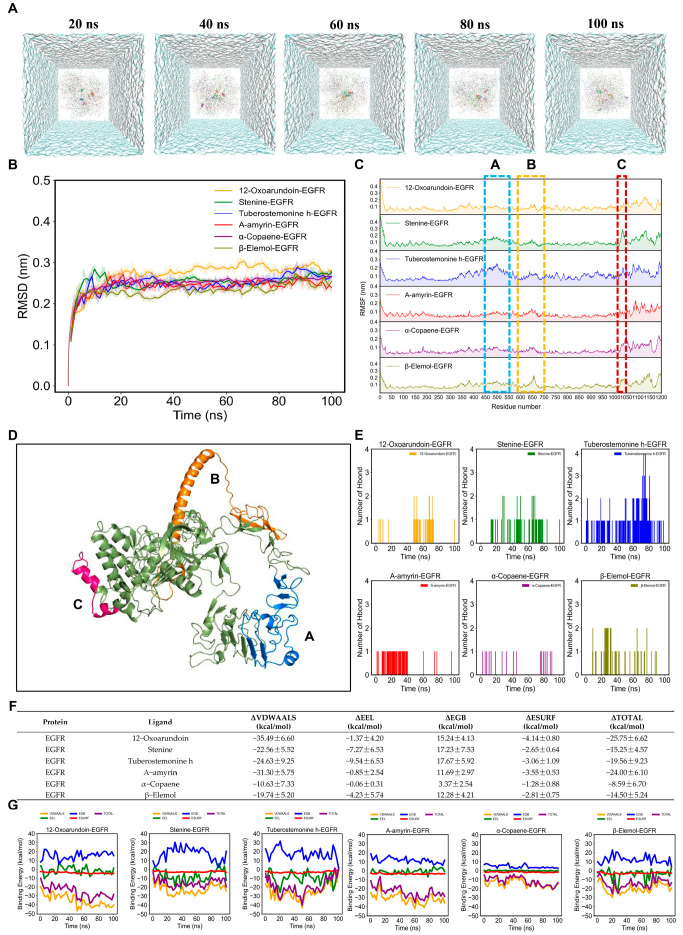
MD simulations of six AMK ingredients with EGFR for 100 ns. (**A**) Simulation trajectories of six AMK ingredients with EGFR in the aqueous solution system for 100 ns. (**B**) RMSD plot of AMK ingredients with EGFR for 100 ns. (**C**) RMSF plot of AMK ingredients with EGFR for 100 ns. (**D**) High-flexibility amino acids (potentially reactive residues). (**E**) The number of AMK hydrogen bonds with EGFR for 100 ns. (**F**,**G**) Binding-free energies of AMK ingredients with EGFR for 100 ns. ΔGGAS = ΔVDWAALS + ΔEEL; ΔGSOLV = ΔEGB + ΔESURF; ΔTOTAL = ΔGGAS +ΔGSOLV. *EGFR*—Epidermal Growth Factor Receptor. ΔEEL—Electrostatic Interaction Energy ΔESURF—Nonpolar Solvation Energy, ΔEGB—Polar Solvation Energy.

The simulation trajectories of six AMK ingredients with EGFR for 100 ns were presented in Figure 7A. In Figure 7B, the root main square deviation (RMSD) value of six AMK ingredients with EGFR was smaller than 0.1 nm after 30 ns. The RMSF values were higher in the 450–550 residues, 600–700 residues, and 1020–1150 residues within the EGFR protein backbone (Figure 7C). The high-flexibility amino acids are shown in Figure 7D. The active ingredients of AMK may bind to the residues within the pocket, suggesting a strong interaction with EGFR. The ranking of the number of hydrogen bonds in six ingredients of AMK with EGFR is as follows: tuberostemonine h-EGFR > stenine-egfr > β-elemol-EGFR > 12-oxoarundoin-EGFR > α-amyrin-EGFR > α-copaene-EGFR (Figure 7E). In molecular dynamics simulations, free energy calculations are commonly used to evaluate the interaction energies between protein-ligand or protein-protein complexes. The binding free energy primarily consists of two components: gas-phase free energy (ΔGGAS) and solvation free energy (ΔGSOLV). The gas-phase free energy includes van der Waals interaction energy (ΔVDWAALS) and electrostatic interaction energy (ΔEEL), which reflect the direct interactions between molecules. Solvation free energy is composed of polar solvation energy (ΔEGB) and nonpolar solvation energy (ΔESURF), which describe the contributions of solvent to the solute’s polar interactions and hydrophobic effects, respectively. Finally, the total binding free energy (ΔTOTAL) is obtained by integrating these two components, providing insights into the binding stability of ligands or proteins under physiological conditions. For systems of six AMK ingredients with EGFR, ΔVDWAALS, and ΔESURF predominantly contributed to binding-free energy, as displayed in Figure 6F,G. However, ΔEEL and ΔEGB showed a minor contribution to the binding-free energy. Ultimately, the ranking of the ΔTOTAL within systems of six AMK ingredients with EGFR was as follows: α-copaene-EGFR > β-elemol-EGFR > stenine-EGFR > tuberostemonine h-EGFR > α-amyrin-EGFR > 12-oxoarundoin-EGFR (Figure 7F,G). A lower ΔTOTAL rank indicated a stronger affinity between the ligand and the protein. Therefore, 12-Oxoarundoin exhibited the strongest affinity with EGFR.

**Figure 8:**
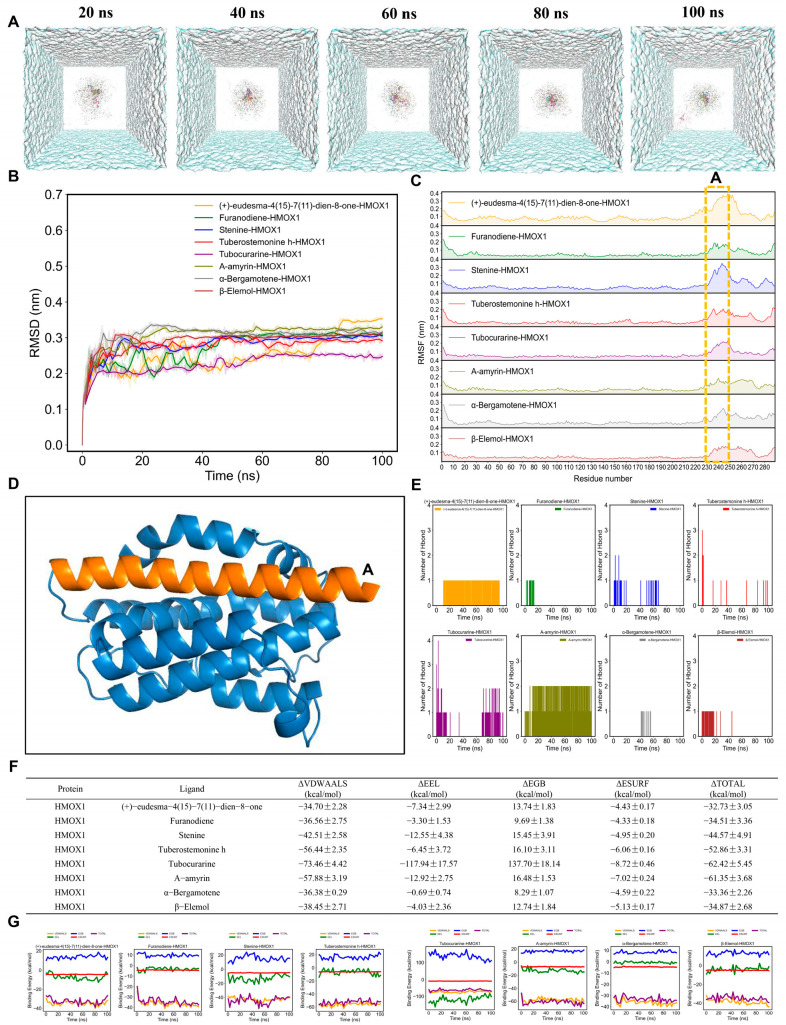
MD simulations of eight AMK ingredients with HMOX1 for 100 ns. (**A**) Simulation trajectories of eight AMK ingredients with HMOX1 in the aqueous solution system for 100 ns. (**B**) RMSD plot of AMK ingredients with HMOX1 for 100 ns. (**C**) RMSF plot of AMK ingredients with HMOX1 for 100 ns. (**D**) High-flexibility amino acids (potentially reactive residues). (**E**) The number of AMK hydrogen bonds with HMOX1 for 100 ns. (**F**,**G**) Binding-free energies of AMK ingredients with HMOX1 for 100 ns. ΔGGAS = ΔVDWAALS + ΔEEL; ΔGSOLV = ΔEGB + ΔESURF; ΔTOTAL = ΔGGAS +ΔGSOLV. *HMOX1*—Heme Oxygenase-1, ΔEEL—Electrostatic Interaction Energy ΔESURF—Nonpolar Solvation Energy, ΔEGB—Polar Solvation Energy.

Furthermore, the simulation trajectories of eight AMK ingredients with HMOX1 for 100 ns were presented in Figure 8A. The RMSD values of eight AMK ingredients with HMOX1, except (+)-eudesma-4(15)-7(11)-dien-8-one, were smaller than 0.1 nm, ranging from 50 to 100 ns (Figure 8B). The RMSF values of the HMOX1 backbone within each complex showed similar fluctuation trends (Figure 8C). Furthermore, the RMSF values were higher in 230–250 residues (Figure 8D). The active ingredients of AMK may bind to these residues within the pocket, suggesting a strong interaction with HMOX1. For the eight AMK ingredients with HMOX1, the ranking of the number of hydrogen bonds is as follows: α-bergamotene-HMOX1 > furanodiene-HMOX1 > β-elemol-HMOX1 > α-amyrin-HMOX1 > (+)-eudesma-4(15)-7(11)-dien-8-one-HMOX1 > Stenine-HMOX1 > tuberostemonine h-HMOX1 > tubocurarine-HMOX1 (Figure 8E). In addition, the ΔVDWAALS and ΔEEL values made a higher contribution to the binding-free energy than systems with six AMK ingredients with EGFR (Figure 7F,G). Eventually, the ranking of the ΔTOTAL within systems with eight AMK ingredients with HMOX1 is as follows: (+)-eudesma-4(15)-7(11)-dien-8-one-HMOX1 > α-bergamotene-HMOX1 > furanodiene-HMOX1 > β-elemol-HMOX1 > stenine-HMOX1 > tuberostemonine h-HMOX1 > α-amyrin-HMOX1 > Tubocurarine-HMOX1 (Figure 8F,G). A lower rank of ΔTOTAL indicated a stronger affinity between the ligand and the protein. Therefore, Tubocurarine exhibited the strongest affinity with HMOX1.

### 3.7. Zebrafish Model Validation

**Figure 9:**
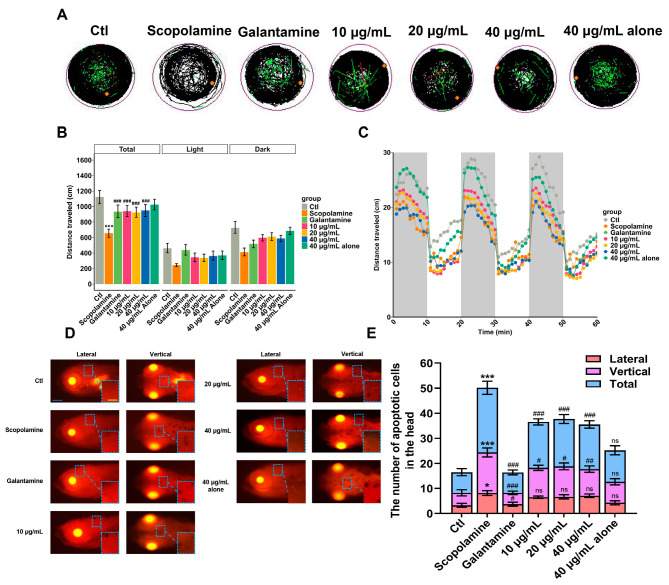
Locomotion analysis and apoptosis assessment. (**A**) Representative images of digital tracks. The black lines indicate slow-speed movement; green lines are linked to medium-speed movement; red lines are associated with high-speed movement; orange dots represented the initial position. (**B**) The total distance traveled by zebrafish exposed to different concentrations of AMK at 6 dpf. (**C**) Velocity in light–dark challenge, which contained three cycles of light–dark phases, lasting 10 min for each phase. (**D**,**E**) Representative dorsal and lateral views of the zebrafish brain showing apoptotic cells within the brain; scale bar: 200 μm. Quantification of apoptotic cells in the zebrafish brain; each group (*n* = 6). * *p* < 0.05; ** *p* < 0.01 *** *p* < 0.001; ns—not significant; vs. Ctl. ^#^ *p* < 0.05; ^##^ *p* < 0.01; ^###^ *p* < 0.001 vs. AD. AD—Alzheimer’s Disease Group, Ctl—Control Group.

The zebrafish locomotor behavior may reflect AD-related characteristics such as cognitive- and motor-slowing. Compared to the AD group (scopolamine), the positive drug group (galantamine) and AMK treatment group showed an increased swimming distance in zebrafish under alternating light–dark conditions, with no significant difference in the maximum concentration compared to the control group (Figure 9A,B). After AMK treatment at 6 dpf, the swimming speeds of zebrafish in all groups under changing light–dark environments are shown in Figure 9C. Compared to the AD group, the positive drug group and the AMK group exhibited increased average swimming speeds and enhanced responsiveness to sudden light stimuli (Figure 9C). The above results indicate that AMK significantly alleviated AD-like motor slowing, demonstrating neuroprotective effects across different concentration groups.

The pathogenesis of AD is closely related to cell apoptosis. Therefore, a TUNEL staining experiment was conducted to observe the apoptotic status of zebrafish brain cells. The results are shown in Figure 9D, where apoptotic cells appear as bright dots that are clearly visible in both dorsal and lateral views of the zebrafish. According to the statistical results of apoptotic cell counts in the brain region presented in Figure 9E, the number of apoptotic cells in the AD group zebrafish significantly increased compared to the blank control group. In contrast to the AD group, the positive drug group and AMK groups showed a significant reduction in the number of apoptotic cells, indicating that AMK has a mitigating effect on apoptosis in zebrafish brain cells.

**Figure 10:**
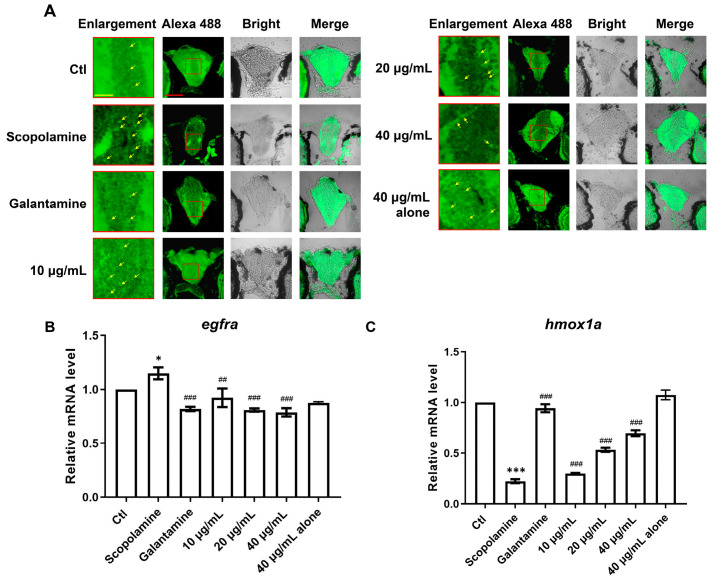
The deposition of Aβ in the brain and mRNA expression of two core targets (*egfra* and *hmox1a*). (**A**) Thioflavin S specifically stains Aβ plaques, with red squares indicating magnified areas and yellow arrows marking pathological plaques; scale bar: 60 μm. (**B**,**C**) The bar graphs display the fold changes in mRNA expression for *egfra* and *hmox1a*. Data are presented as mean ± sem and were analyzed via one-way ANOVA, with *n* = 3. * *p* < 0.05; *** *p* < 0.001; vs. Ctl. ^##^ *p* < 0.01; ^###^ *p* < 0.001 vs. AD. *EGFR*—Epidermal Growth Factor Receptor, *HMOX1*—Heme Oxygenase-1. Ctl—Control Group.

The primary neuropathological feature of AD patients is the abnormal accumulation of Aβ, leading to the formation of extracellular amyloid plaques, which affect, damage, and even lead to the apoptosis of brain cells. Thioflavin S is a fluorescent histochemical marker that specifically labels the dense core region of Aβ plaques. The results of Thioflavin S staining in this experiment are shown in Figure 10A, where the red box indicates the magnified area of the brain, and the red arrow points to the Aβ plaques. In contrast to the AD group, both the positive drug group and the AMK group showed a marked reduction in the number of Aβ plaques in the brain, indicating that AMK can improve the abnormal pathological accumulation of Aβ in the zebrafish brain.

Based on the computational results, we speculated that AMK may improve AD-like symptoms via *EGFR* and *HMOX1*. Therefore, we assessed the expression levels of *egfra* and *hmox1* genes in zebrafish. Compared to the control group, *egfra* expression was significantly upregulated in the AD group, consistent with the findings from TWAS, SMR, and MR analyses, and this abnormal expression was significantly reversed by both AMK and the positive drug (Figure 10B). Additionally, *hmox1a* expression was significantly downregulated in the AD group compared to the control group, consistent with the TWAS, SMR, and MR results, and this abnormal expression was significantly reversed through AMK and the positive drug (Figure 10C). Thus, AMK ameliorated AD-like symptoms through the modulation of *EGFR* and *HMOX1*.

## 4. Discussion

Related research indicated that ferroptosis significantly contributes to the progression of AD. Lipid peroxidation is a key mechanism in ferroptosis. Additionally, AMK possesses neuroprotective effects and exhibits antioxidant activity. Thus, it is proposed that AMK may ameliorate AD-like symptoms through targeting ferroptosis-related pathways, potentially inhibiting ferroptosis through the suppression of lipid peroxidation. This study identified ferroptosis-related targets modulated by AMK for AD treatment and employed a zebrafish model to investigate the amelioration of AD-like symptoms via AMK, as well as to validate the expression of ferroptosis-related targets.

In the brain datasets, significant differences were observed in the ferroptosis, phosphorylation, and apoptosis pathways, implying that ferroptosis exacerbates tau protein phosphorylation and apoptosis in the brain. In the blood datasets, we found significant differences in ferroptosis, lipid peroxidation, phosphorylation, and apoptosis pathways. This indicated that ferroptosis not only affected the brain but also exhibited systemic effects through blood-associated pathways. The absence of lipid peroxidation in the brain tissue may be attributed to the higher antioxidant capacity and protective function of the blood–brain barrier [58], which mitigate the impact of oxidative stress on brain lipids. In contrast, blood, which lacks similar protective mechanisms and is rich in polyunsaturated fatty acids susceptible to peroxidation, is more prone to lipid peroxidation [59]. Notably, ferroptosis exhibited the most significant pathway activity in both the blood and brain datasets, indicating its specific relevance to AD. Consistent with previous studies, several biomarkers of AD pathogenesis correlate with ferroptosis characteristics, including increased iron accumulation, elevated lipid peroxides and reactive oxygen species (ROS), and decreased levels of glutathione GSH and glutathione peroxidase 4 GPX4 [60].

We identified 60 ferroptosis-related target genes in the brain and 18 ferroptosis-related target genes in the blood. According to related studies, there is a close connection between blood circulation and the brain, with blood biomarkers and metabolic products reflecting pathological changes in the brain [61]. Furthermore, the hippocampus is a brain region that is strongly associated with worse cognition. The early pathological changes in AD often occur in the hippocampal region [62,63]. Hence, these targets may be potential targets for AD treatment. To identify potential ferroptosis-related target genes, logistic regression was used with seven FS methods. LassoCV method with logistic regression was the best-performing method (F1 = 0.83 ± 0.11, AUC = 0.85 ± 0.10) in the GSE5281 dataset and showed a promising generalization capacity in brain datasets. Therefore, these features are reliable as targets. Moreover, the SVMCV method with logistic regression is the best-performing method (F1 = 0.58 ± 0.07, AUC = 0.64 ± 0.06) in integrated blood GSE63060 and GSE63061 datasets and demonstrated a degree of generalization ability in blood datasets. Compared with the potential target blood genes, the potential target genes in the brain exhibited higher and more stable AUC values. The hippocampus is essential for memory formation and spatial navigation [64]. Notably, confounding variables might affect blood biomarkers, thereby compromising their accuracy and reliability, such as age, sex, the presence of comorbidities, and lifestyle factors [65]. Due to these reasons, biomarkers from brain tissue exhibited better discriminatory power than blood biomarkers. Accordingly, the results of attention mechanism based deep learning model showed improvements in the brain datasets and a limited improvement in the blood datasets. Finally, brain biomarkers are a better input than blood biomarkers in the attention mechanism-based deep learning model.

To investigate the relationship between target genes and AD risk from the perspective of genetic association studies, a TWAS, SMR, and MR were performed. *EGFR* and *HMOX1* were significantly associated with AD risk. In addition, *EGFR* and *HMOX1* were prioritized as drug target genes in the brain and blood, respectively. EGFR, the founding member of the receptor tyrosine kinase superfamily, plays essential roles in embryogenesis and adult tissues, including in growth, differentiation, and tissue maintenance and repair [66]. Furthermore, the results of Phe-WAS analysis showed that the potential target genes (*EGFR* and *HMOX1)* had no side effects in other systems. According to previous studies, EGFR was highly expressed in the hippocampus. Consistent with this study, *EGFR* was screened from the transcriptome in the hippocampus and prioritized in the brain cis-eQTL with AD GWAS [67]. In AD mouse models, EGFR inhibitors demonstrated efficacy in reducing Aβ pathology and enhancing cognitive function [68]. Additionally, the overexpression of EGFR was related to destroyed autophagy and ferroptosis [69]. Thus, EGFR is a potential target associated with ferroptosis for AD treatment. The GSEA results indicated that *EGFR* may be involved in oxidative phosphorylation-related pathways, inhibiting lipid peroxidation and suppressing ferroptosis, which results in reduced neuronal apoptosis in the brain. Additionally, it may decrease tau protein phosphorylation via AMPK-mediated phosphorylation [70], leading to a reduction in Aβ accumulation. HMOX1 is an enzyme involved in heme degradation, producing Fe2^+^, CO, and biliverdin/bilirubin [71]. It is linked to the pathological features of AD, including neurofibrillary tangles and senile plaques [72]. Moreover, there is limited research indicating the relationship between HMOX1 and ferroptosis in AD. In this study, *HMOX1* was identified as a potential target for AD treatment, providing further evidence supporting the effect of HMOX1 on AD. The GSEA results indicated that *HMOX1* may be involved in the lysosome and phagosome pathways. *HMOX1* can regulate iron metabolism and oxidative stress in the blood [73], thereby inhibiting ferroptosis. By promoting iron regulation and exerting antioxidative effects, *HMOX1* alleviates oxidative stress, which in turn activates autophagy to remove harmful intracellular substances [74]. This mechanism reduces neuronal apoptosis and Aβ accumulation in the brain, thereby combating the progression of neurodegenerative diseases. Therefore, drug development targeting EGFR and HMOX1 may be promising for AD treatment.

To validate the anti-AD activity of AMK, three molecular docking strategies and molecular dynamics simulations were conducted. The combination of three molecular docking algorithms can help to eliminate false positives. The results of the molecular docking indicated that there are six ingredients that bind with EGFR and eight ingredients that bind with HMOX1. A drug-likeness assessment provided further insights into the drug-like potential and biological activity of the AMK ingredients. The drug-likeness properties implied that all AMK ingredients adhered to Lipinski’s rule of five. All AMK ingredients exhibited good potential for developability, indicating that these ingredients may have promising therapeutic properties or applications. The molecular dynamics simulation results indicated that the key ingredients of AMK could bind with EGFR and HMOX1. Notably, the RMSD values of eight AMK ingredients were more stable than those of the six AMK ingredients with EGFR. Similarly, Tubocurarine with HMOX1 exhibited the lowest binding-free energy in the AMK ingredients in the HMOX1 system.

In the molecular dynamics simulation, the high RMSF values of the EGFR backbone were observed in residues 600–700, particularly in the region of amino acids 688–704, which are involved in dimerization and phosphorylation [75]. This suggested that the active ingredients of AMK may regulate ferroptosis by interacting with the kinase domain of EGFR. In the binding process between AMK and HMOX1, high values were observed in residues 230–250. Related studies suggested that the polar amino acids in regions 227–248 may regulate oxidative stress and lipid peroxidation, thereby inhibiting ferroptosis [76,77].

We speculated that the active ingredients of AMK may bind to the kinase domain of EGFR and inhibit protein phosphorylation, thereby affecting Aβ accumulation. Phosphorylation is typically associated with the formation of amyloid plaques. Therefore, the inhibitory effect of AMK on phosphorylation may slow down or modify Aβ accumulation, influencing the progression of AD. Meanwhile, the active AMK ingredients may also bind to the catalytic domain of HMOX1 to inhibit lipid peroxidation and modulate a dysregulated metabolism, ultimately reducing neuronal apoptosis in the brain.

Our study indicated that AMK alleviated AD-like motor-slowing and reduced brain plaque formation and cell apoptosis in zebrafish. This revealed that AMK may improve AD-like symptoms, with potential therapeutic significance. The qPCR results showed that *egfra* and *hmox1a* were abnormally expressed in the AD group. AMK reversed these abnormal mRNA expressions, and at its maximum concentration, showed no toxicity, consistent with the computational chemistry results, suggesting that AMK may alleviate AD-like symptoms via EGFR and HMOX1.

## 5. Conclusions

By combining multi-omics, computational chemistry, and a zebrafish AD model, we found that AMK significantly relieved AD-like symptoms by modulating the target genes *EGFR* and *HMOX1*, which implied that AMK has anti-AD effects via inhibiting ferroptosis through alterations in EGFR and HMOX1. These findings highlight the therapeutic potential of AMK as a promising candidate for AD treatment through ferroptosis regulation.

## Figures and Tables

**Table 1:** Results of Mendelian randomization analysis.

Exposure	Outcome	Method	*p*	OR	OR (95% LCI)	OR (95% UCI)
*EGFR*	GCST007320	MR Egger	1.28 × 10^−5^	1.014	1.009	1.019
*EGFR*	GCST007320	Inverse variance weighted	1.07 × 10^−25^	1.014	1.011	1.016
*EGFR*	GCST007320	Weighted median	4.31 × 10^−13^	1.013	1.01	1.017
*EGFR*	ukb-b-323	MR Egger	1.20 × 10^−4^	1.003	1.001	1.004
*EGFR*	ukb-b-323	Inverse variance weighted	2.56 × 10^−22^	1.003	1.002	1.003
*EGFR*	ukb-b-323	Weighted median	1.81 × 10^−12^	1.003	1.002	1.004
*HMOX1*	GCST007320	MR Egger	3.53 × 10^−8^	0.974	0.966	0.982
*HMOX1*	GCST007320	Weighted median	6.52 × 10^−7^	0.987	0.982	0.992
*HMOX1*	GCST007320	Inverse variance weighted	3.07 × 10^−6^	0.992	0.988	0.995
*HMOX1*	GCST007320	MR Egger	3.53 × 10^−8^	0.974	0.966	0.982
*HMOX1*	GCST007320	Weighted median	4.12 × 10^−7^	0.987	0.982	0.992
*HMOX1*	GCST007320	Inverse variance weighted	3.07 × 10^−6^	0.992	0.988	0.995

*EGFR*—Epidermal Growth Factor Receptor, *HMOX1*—Heme Oxygenase-1.

**Table 2:** Heterogeneity test of Mendelian randomization results.

Exposure	Outcome	Method	Q	Q df	Q *p*
*EGFR*	GCST007320	MR Egger	16.172	27	0.950
*EGFR*	GCST007320	Inverse variance weighted	16.174	28	0.963
*EGFR*	ukb-b-323	MR Egger	22.455	27	0.714
*EGFR*	ukb-b-323	Inverse variance weighted	22.643	28	0.751
*HMOX1*	GCST007320	MR Egger	14.444	24	0.936
*HMOX1*	GCST007320	Inverse variance weighted	24.520	25	0.490
*HMOX1*	ukb-b-323	MR Egger	37.865	59	0.985
*HMOX1*	ukb-b-323	Inverse variance weighted	45.509	60	0.917

*EGFR*—Epidermal Growth Factor Receptor, *HMOX1*—Heme Oxygenase-1, MR—Mendelian Randomization.

**Table 3:** Horizontal pleiotropy test of Mendelian randomization results.

Exposure	Outcome	Egger Intercept	SE	*p*
*EGFR*	GCST007320	0.000	0.001	0.962
*EGFR*	ukb-b-323	0.000	0.000	0.669
*HMOX1*	GCST007320	0.005	0.002	0.351
*HMOX1*	ukb-b-323	0.001	0.000	0.282

*EGFR*—Epidermal Growth Factor Receptor, *HMOX1*—Heme Oxygenase-1.

## Data Availability

The datasets analyzed in this study are available on the GEO (https://www.ncbi.nlm.nih.gov/geo/, accessed on 3 February 2025), GWAS Catalog (https://www.ebi.ac.uk/gwas/home, accessed on 3 February 2025), and OpenGWAS project (https://gwas.mrcieu.ac.uk/, accessed on 3 February 2025) websites. Moreover, we uploaded the complete raw data in the Supplementary Materials.

## Supplementary Materials

The following supporting information can be downloaded at: https://www.mdpi.com/article/10.3390/cimb47020118/s1.
