# Supplementary figures and images for: Exploring the Efficacy and Target Genes of Atractylodes Macrocephala Koidz Against Alzheimer’s Disease Based on Multi-Omics, Computational Chemistry, and Experimental Verification
Source: Curr Issues Mol Biol. 2025 Feb 11;47(2):118. doi: 10.3390/cimb47020118 (PMC11853862; doi:10.3390/cimb47020118)

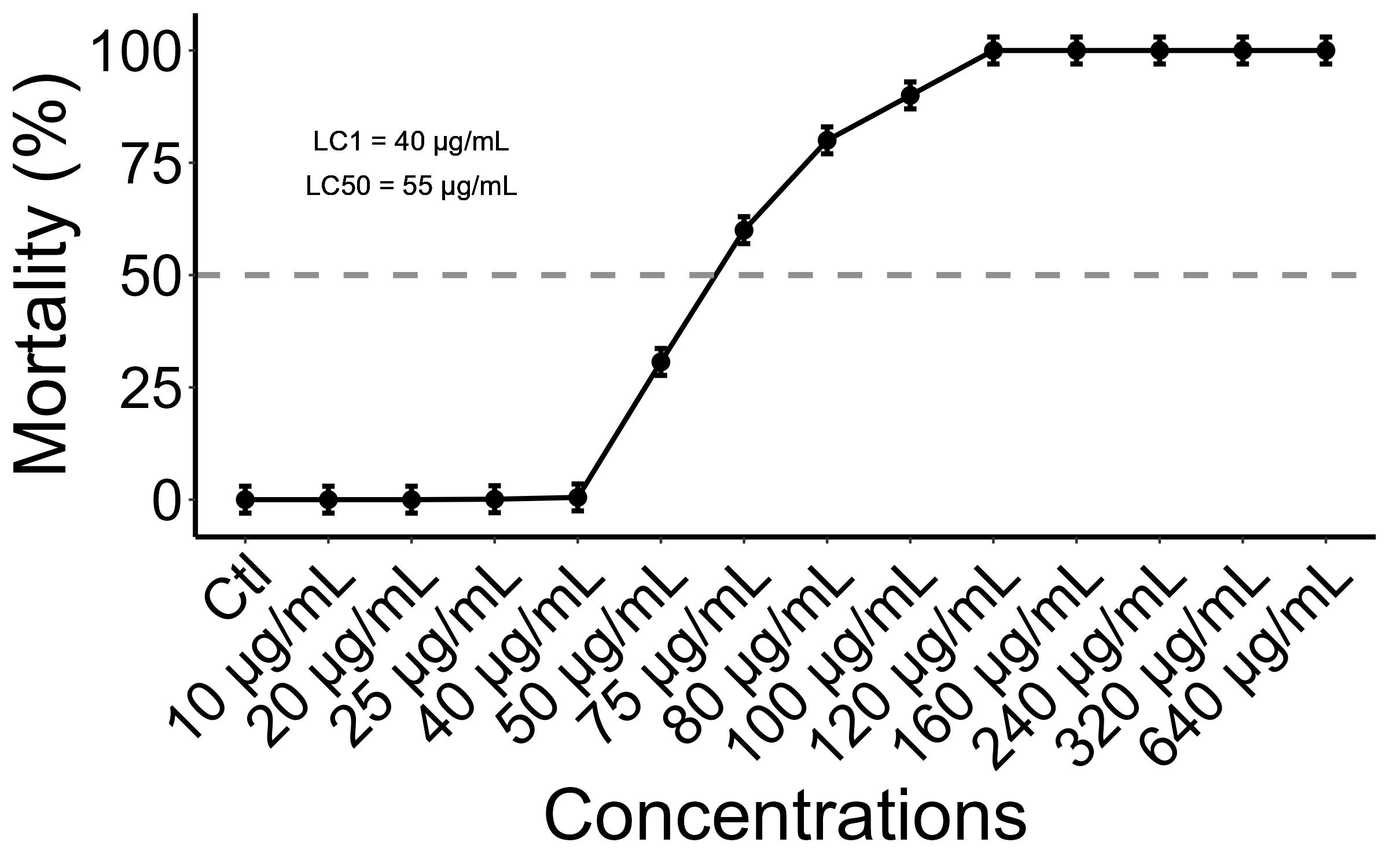

Supplement: Supplementary file 1 [file cimb-47-00118-s001.zip › Supplementary Figure S1.jpg]
